# Winemaking Byproducts as Source of Antioxidant Components: Consumers’ Acceptance and Expectations of Phenol-Enriched Plant-Based Food

**DOI:** 10.3390/antiox9080661

**Published:** 2020-07-24

**Authors:** Cristina Proserpio, Giovanna Fia, Ginevra Bucalossi, Bruno Zanoni, Sara Spinelli, Caterina Dinnella, Erminio Monteleone, Ella Pagliarini

**Affiliations:** 1 Department of Food, Environmental and Nutritional Sciences (DeFENS), University of Milan, 20133 Milan, Italy; cristina.proserpio@unimi.it; 2 Department of Agricultural, Food, Environmental and Forestry (DAGRI), University of Florence, 50144 Firenze, Italy; giovanna.fia@unifi.it (G.F.); ginevra.bucalossi@unifi.it (G.B.); bruno.zanoni@unifi.it (B.Z.); sara.spinelli@unifi.it (S.S.); caterina.dinnella@unifi.it (C.D.); erminio.monteleone@unifi.it (E.M.)

**Keywords:** sustainability, phenols, unripe grapes, taste, food preferences

## Abstract

One of the food industry’s priorities is to recover byproducts and move towards more sustainable systems. Among wine-chain byproducts, unripe grapes represent a promising source of antioxidants. However, the development of new foods enriched using phenol-rich ingredients is challenging due to their sensory attributes. The aims of the present study were to (1) use phenol-rich extract from unripe grapes to enrich a model plant-based food (beetroot puree—BP); (2) evaluate consumers’ acceptance and expectations for the beetroot purée samples. The effect of information about the sustainability and pro-health activity of value-added ingredients on consumers’ responses was also investigated. Four beetroot purees with increasing concentrations of phenol extract (0–1.93 g/kg) added were evaluated by 101 participants in three tasting conditions (blind: only samples; expected: only information without tasting; real: both samples and information).Liking slightly decreased with increasing concentrations of phenol extract, even if all the samples were considered acceptable. The health and sustainability information increased the hedonic expectations, although it was not assimilated by all consumers involved. The development of new phenol-enriched foods using functional ingredients from unripe grapes is challenging. However, it is also promising, since all the samples were generally accepted by the consumers and they presented phenol levels that were stable over time and that could have positive health effects when consumed.

## 1. Introduction

Recently, great interest and various efforts have been addressed towards sustainability issues. One of the food industry’s priorities is to use side-streams from food-production chains, moving towards a system that pays more attention to both the environment and consumers’ health. Several byproducts from the food-production chain, especially from fruit and vegetable processing, are today considered cheap and important sources of valuable and functional components. For example, dehulling of rice and grinding of wheat yield byproducts like straw or bran, which are rich in highly nutritious proteins as well as dietary fiber. Potato peels and processing wastewater have also been extensively studied for phenol extraction [1]. In this context, modern technologies allow the recovery of specific compounds and their recycling inside the food-production chain as value-added ingredients to develop new food products with added market value [1,2]. These functional compounds could also play an important role in view of the necessity of providing accessible and healthy food for the growing population in the next century [3].

Among these compounds, phenols from byproducts of plant-based food industry present antioxidant, antimicrobial, anti-inflammatory, and chemo-preventive properties and can be used as new value-added food ingredients [4]. In particular, phenols and carotenoids from fruit byproducts can be used to lengthen the shelf lives of products by delaying the development of off-flavors and rancidity [5,6]. Recently, phenol extracts from olive mill wastewater were successfully used to increase the antioxidant activity of plant-based foods with different macro-compositions [7].

Unripe grapes discarded during thinning operations in the high-quality-wine production chain represent another interesting but scarcely investigated source of phenol compounds [8]. Similarly to other byproducts of wine industry, these unripe grapes contain valuable nutrients for the human diet, such as sugars, organic acids, mineral salts, dietary fiber, vitamins, and phenols [9,10]. However, the development of new foods with added phenol extract from unripe grapes is challenging, since these phenols are characterized by specific sensory attributes, such as sourness and astringency [8,11,12], that could negatively impact the sensory properties of the food matrices into which the extracts are added. Indeed, it has been reported that the addition of grape-skin powders to soft cow-milk cheeses or apple purees resulted in a decrease in liking scores, especially with increasing powder amounts [13,14]. Hence, it is important to consider the consumers’ responses to these new foods, since foods’ market success is mainly guided by food preferences and consumers are not actually inclined to compromise their taste for healthiness [15].

Product information about positive health effects has been reported to be highly influential in affecting consumers’ expectations [16,17,18,19]. However, this information is not always enough to lead consumers to accept and consume new food products. Indeed, an extensive discrepancy between attitudes, behaviors, and values has been reported, since consumers often establish behaviors and habits in contrast with their beliefs, intentions, and opinions [20,21].

In the present study, beetroot puree was used as model food to which was added increasing concentrations of phenol-rich extract obtained from grapes gathered thinning operations, since this extract is characterized by an intense purple color. Moreover, as recently demonstrated by Bucalossi et al. [8], beetroot is characterized by an intense sweet taste that may counterbalance the sourness of the phenol extract. Therefore, after several preliminary tests, beetroot was considered the most suitable choice in terms of visual and tasting cues. 

The aims of the present study were to (1) use phenol-rich extract from unripe grapes to enrich a model plant-based food (beetroot puree—BP); (2) evaluate consumers’ acceptance and expectations for the beetroot puree samples. In particular, the effect of information about sustainability and the pro-health activities of functional ingredients on consumers’ responses was investigated. The relationship between consumers’ health and taste attitudes and acceptance of the developed food formulations was also assessed.

## 2. Materials and Methods

### 2.1. Unripe Grape (UG) Extract Preparation and Analysis

#### 2.1.1. Phenol Extract Preparation

The unripe grapes, cv Merlot, were thinned in August 2017, in a vineyard situated in Velletri, Rome, Italy. The UG extract was obtained following the method described by Bucalossi et al. [8]. The unripe grapes were crushed and macerated for 72 h at 6 °C, with the addition of dry ice. After sedimentation and decantation, the extract was filtered and the sugar was eliminated by ultrafiltration performed with a spiral-wound configuration membrane, at a cut-off of 2500 Dalton (General Electric, Boston, Massachusetts, United States). The juice was lyophilized with gum arabic (2% w/v) (Nexira Food, Rouen Cedex, France) as support, and the powder was stored under vacuum in a desiccator at room temperature, in the dark. 

Phenol extracts were prepared following the method described by Turkmen et al. [22]. One gram of phenol-enriched plant-based food (beetroot puree) was added to 4.5 mL of 80% aqueous methanol solution prepared with methanol (Carlo Erba Milan, Italy) and homogenized in a shaker for 2 h. The mixture was centrifuged at 13,440× *g* for 15 min at room temperature and the supernatant decanted in polypropylene tubes. These steps were carried out twice. The supernatant was filtered with Whatman No 1 filter paper. The extraction procedure was performed in triplicate.

#### 2.1.2. Determinations of pH and Total Acidity

Determinations of pH and total acidity were carried out following the analytical method described by the International Organization of Vine and Wine (OIV) (International Organization of Vine and Wine Website, 2017) [23]. 

#### 2.1.3. Total Phenol Content 

The total phenol (TP) content was evaluated by Folin–Ciocalteau assay [24]. A volume of 1 mL of each sample was added to 4 mL of sodium carbonate (10% w/v) and shaken well; after 5 min, 1 mL of Folin–Ciocalteau reagent was added to the mixture. After 90 min at the dark, the absorbance at 700 nm was measured with a Perkin Elmer Lamba 10 spectrophotometer (Waltham, MA, USA). A calibration curve was obtained with (+)-catechin solution (concentration range 5–500 mg/L) and the results were expressed as mg of (+)-catechin/L of UG solution or kg of beetroot puree. Samples were analyzed in triplicate. The standards and reagents were purchased from Sigma-Aldrich (Milan, Italy).

#### 2.1.4. Antioxidant Activity

The antioxidant activity (AA) was evaluated according to the method reported by Brand-Williams et al. [25]. Briefly, a quantity of 0.236 g of DPPH^•^ was dissolved in 100 mL of methanol to obtain a standard solution of DPPH^•^ (6 × 10^−5^ M). For the reaction, a volume of 0.1 mL of each sample was added to 3.9 mL of DPPH^•^ solution. For the reference sample, 0.1 mL of methanol was mixed with 3.9 mL of DPH solution. The free-radical-scavenging activity was evaluated by measuring the absorbance at 515 nm after 30 min of reaction at 30 °C using a Perkin Elmer Lambda 10 spectrophotometer (Waltham, MA, USA). The results were expressed as µmol Trolox equivalent (TE)/L of UG solution or kg of beetroot puree. A calibration curve was obtained with Trolox solution (concentration range 10–600 µmol/L). The assay was carried out in triplicate. All chemicals were purchased from Sigma-Aldrich (Milan, Italy).

#### 2.1.5. LC-HRMS Analysis 

Analysis of the phenolic compounds and glutathione was carried out via liquid chromatography–high-resolution mass spectrometry (LC-HRMS) according to Fia et al. [10], using an Accela 1250 (Thermo Fisher Scientific, Waltham, MA, USA) coupled with a Linear Trap Quadrupole (LTQ) OrbitrapExactive mass spectrometer (Thermo Fisher Scientific, Waltham, MA, USA) equipped with an electrospray ionization (ESI) source in negative mode. Peak assignment was carried out on the basis of the exact mass values of the molecular ions and the cis and trans forms were recognized by comparison of the retention times with the standard sample. The standards were purchased from Sigma-Aldrich (Milan, Italy), except for the quercetin 3-*O*-glucoside, which was supplied by Analytik GmbH (Rülzheim, Germany). Coumaric and ferulic acids were used as standards for coutaric and fertaric acids due to the lack of reference materials. Data were expressed as mg of phenols/kg of the UG extract or beetroot sample. The phenols recovered (recovery %) from the functionalized beetroot samples were calculated as the percentage of the sum of UG phenols added plus phenols measured in the beetroot puree before the addition.

### 2.2. Consumer Test

#### 2.2.1. Participants

One hundred and one participants (59 women and 42 men; mean age: 22.65 ± 0.23) were recruited from the students and staff of the Faculty of Agronomical and Food Sciences of the University of Milan (Italy). The exclusion criteria were: subjects who did not like beetroots, pregnant women, subjects suffering from food intolerances and allergies, and those who were on medical treatments that might modify taste perception. This study was conducted in compliance with the principles laid down in the Declaration of Helsinki, and all subjects provided informed written consent prior to participation. 

#### 2.2.2. Sample Preparation

Beetroot functionalized samples were prepared according to Bucalossi et al. [8]. In brief, beetroot puree (BP) was prepared by blending at maximum speed 500 g of peeled steamed beetroots for about 1 min, using a Kenwood FDM 780 mixer (Kenwood, Treviso, Italy), until a homogeneous product was obtained.

The extract was diluted in distilled water to obtain four solutions with 0.00, 1.45, 3.92, and 6.81 g/L phenol concentrations. These solutions were stored at room temperature in a tightly closed container protected from light and used within 10 h. Beetroots (Ghisetti Srl, Rome, Italy) were used to prepare a puree (nutritional composition per 100 g: energy 44 kcal, protein 2.3 g, carbohydrates 7.2 g, fat 0.1 g, salt 0.2 g). Phenol extract was added to produce four samples at different concentrations: 0.00 (B_0_), 0.41 (B_1_), 1.11 (B_2_), 1.93 (B_3_) g/kg. Samples were prepared on the day of the session and were presented within 30 min to the participants at room temperature. Approximately 20 g of each sample was presented to the participants in plastic cups labeled with three-digit codes. Water was available for rinsing the palate between the samples.

#### 2.2.3. Questionnaires

##### Consumer’s Knowledge and Acceptance of Functional Foods

A modified version of the questionnaire proposed by Verbeke [26] was applied in order to investigate consumers’ knowledge and acceptance of functional foods. The questionnaire consisted of seven items and each statement offered seven graded response alternatives: “strongly disagree” (score 1), “disagree”, “somewhat disagree”, “neutral”, “somewhat agree”, “agree”, and “strongly agree” (score 7).

##### Health and Taste Attitude Scale (HTAS)

The Italian version of HTAS, previously described by Saba et al., [27], was applied to investigate subjects’ food-related attitudes. This questionnaire consists of three health subscales (a total of 20 items) and three taste subscales (a total of 18 items), and each subscale is composed of an equal number of positively and negatively worded statements [28]. The “Pleasure” subscale was not considered in the present study, since previous results obtained in large population studies demonstrated a very low internal reliability of this subscale in Italian subjects [27,29]. Participants were asked to rank the extent to which each statement corresponded to them, and all items were scored on seven-point Likert scales ranging from “strongly disagree” (score 1) to “strongly agree” (score 7). Each subscale was composed of an equal number of positively and negatively worded statements. After reversing the negatively worded statements, a mean score was calculated for each participant and each subscale.

#### 2.2.4. Experimental Procedure

**Figure 1:**
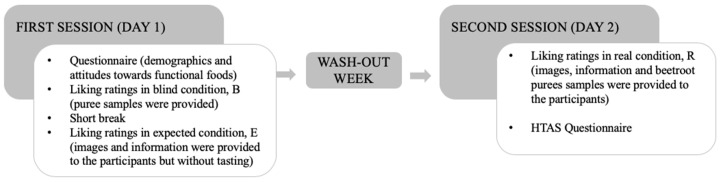
Schematic timeline of the study procedure.

The experimental sessions were conducted in the sensory laboratory at the Department of Food, Environmental and Nutritional Sciences (DeFENS) of the University of Milan (Italy), designed according to ISO guidelines (ISO 8589, 2007). Participants attended two separate test sessions on different days. The participants attended each session at the same time of the day (between 11:30 and 13:30), and had a one week wash-out period between their sessions. They were asked to refrain from eating and drinking anything but water in the 2 h before the test session. Each session took approximately 1 h. The study procedure is reported in Figure 1.

Beetroot puree samples were evaluated under three different tasting conditions: non-informed (blind; B), expected (E), and informed (real; R) conditions according to Deliza and MacFie [30]. At the beginning of each test sessions verbal instructions were provided to the participants about the use of the scales. Liking was measured using a LAM (labeled affective magnitude) scale anchored with the extremes of “greatest imaginable dislike” (rated 0) and “greatest imaginable like” (rated 100) [31] The information provided during the expected condition was as follows: *“From waste and by-products of the wine supply chain, the disposal of which represents a cost for the industries as well as having an impact on the environment, it is possible to recover extracts. These extracts can be used as ingredients for food formulations, increasing the sustainability of the production chain and leading to a possible positive effect on the health of the consumer (for example antioxidant, anticarcinogenic effects, etc.)*”. 

### 2.3. Data Analysis 

#### 2.3.1. Total Phenol Content and Antioxidant Activity of UG Extract

A one-way ANOVA model was used to assess the storage effect on the variations of total phenol content and antioxidant activity of the UG extract. Two-way ANOVA models were used to assess the effect of both phenol concentration and replicates on the antioxidant activity of the enriched food samples and to assess the effects of both the amount of phenol added and replicates on the recovery of UG phenols from enriched foods. The phenols recovered (recovery %) from the functionalized beetroot samples were calculated as the percentage of the sum of UG phenols added plus phenols measured in the beetroot puree before the UG phenol addition.

#### 2.3.2. Liking Assessment under Blind, Expected, and Real Conditions

Firstly, an ANOVA was performed on liking data, considering Sample (B_0_, B_1_, B_2_, and B_3_) and Condition (blind, expected, and real) as fixed factors. Subsequently, two-way ANOVA models were also computed to explore the effect of information on liking scores for each sample. Subjects were considered a random factor. When a significant difference (*p* < 0.05) was found, the Least Significant Difference (LSD) post hoc test was performed as multiple-comparison test. 

Assimilations/contrast effects following negative disconfirmations (blind ratings lower than expected ratings) were independently investigated for each sample, considering the linear regression between individual hedonic disconfirmation (Expected–Blind; E–B) and the Real–Blind difference (R–B) [32,33]. When the slope describing the regression (E–B) vs. (R–B) was positive, an assimilation occurred. When the slope tended to be equal to 1, the assimilation tended to be complete. Slopes lower than 1 revealed an incomplete assimilation. Negative E–B vs. R–B slopes revealed a contrast effect. 

#### 2.3.3. Health and Taste Attitude Scale (HTAS)

Normality checks on the variables measured for the HTAS thorough kurtosis and skewness values confirmed normal univariate distributions. In order to examine the construct validity of the HTAS, the internal consistency reliability of the subscales was explored using Cronbach’s alpha. Correlation coefficients among health and taste subscales were evaluated [34], and the effect of gender on subscale ratings was tested with one-way ANOVA. 

To explore health and taste attitude influences at the individual level on the effect of the information about the phenol content of the beetroot samples on hedonic responses, a map was obtained by means of a principal-component analysis. The matrix included the four samples (B_0_–B_3_) in the three conditions (Blind, Expected, and Real) as lines, and subjects with their liking scores as columns. Samples were included as dummy variables (downweighted in the data matrix) [35]. Consumer clusters were identified via visual interpretation of the correlation-loading plot [36], considering subjects’ positions on the map. A full crossvalidation was computed to validate the interpretation of the first two components. ANOVA models were computed to test the cluster effect on health and taste attitude subscales. 

## 3. Results

### 3.1. Unripe Grape (UG) Extract

Immediately after its production, the UG extract was characterized for total acidity (7.60 ± 0.26 g/L as tartaric acid), pH (3.21 ± 0.02), TP content (20.4 ± 0.9 mg/g), and AA (33.8 ± 0.2 µmol TEAC/g).

The TP content and the AA of the UG extract were evaluated monthly during storage (Table 1). Under the described conditions, the UG extract was stable in term of TP content and AA.

### 3.2. Beetroot Puree Samples

**Figure 2:**
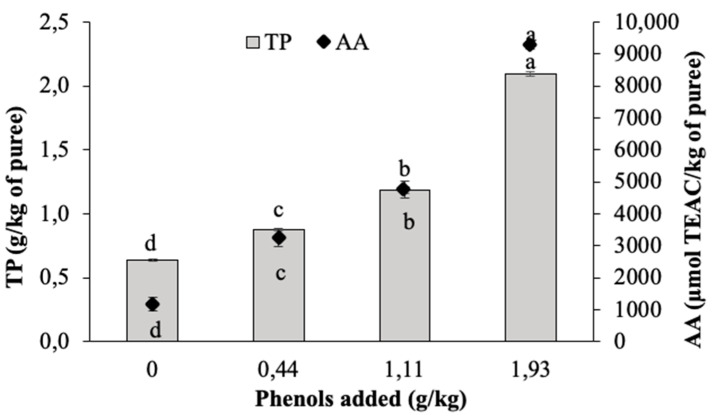
Antioxidant activity and total phenol content of BP enriched with increasing concentrations of UG phenols. The data are the mean of three replicates. The bars represent standard deviation. Different superscript letters represent significant differences (*p* ≤ 0.05).

Increasing amounts of unripe grape phenols (0.00, 0.44, 1.11, 1.93 g/kg) were added to the beetroot purees, and the TP content and AA were determined in the BPs (Figure 2).

The amount of phenols added to the beetroot puree significantly affected the concentration of phenols and the antioxidant activity in the BP (*p* ≤ 0.05). The percentage of total phenols recovered varied significantly depending on the amount of added phenols. The mean value of recovery was 59.6%. The phenol content and the AA detected in the beetroot puree sample without added UG extract can be attributed to the endogenous antioxidant of red beetroot. Pigments such as betalains, and several derivatives of hydroxybenzoic and hydroxycinnamic acids with a wide range of antioxidant activity were previously identified in red beetroot products [37,38,39]. 

Table 2 shows the phenol compositions of the UG extract stock solution (6.81 g/L of phenols), UG extract (powder), beetroot puree, beetroot puree functionalized with the higher phenol concentration (1.93 g/kg), and the percentage of phenols recovered (recovery %) from the functionalized beetroot puree. Twenty phenolic compounds were identified in the UG extract. Phenolic acids were the most abundant class of phenolic compounds detected in the UG extract, and caftaric acid (730.0 mg/kg) accounted for about the 76% of the sum of phenols measured (966.81 mg/kg). Similar results were obtained by Fia et al. [40], who analyzed an extract of unripe grapes (cv. Sangiovese). The observed decrease of ferulic acid content in the functionalized beetroot puree after addition of the UG extract could be due to the esterification reaction with tartaric acid of the unripe grape extract [41,42]. Amounts of trans-resveratrol (31.12 mg/kg), 2-*S*-glutathionyl fertaric acid (18.63 mg/kg), quercetin (14.33 mg/kg), and (+)-catechin (14.16 mg/kg) were higher than those of the remaining phenol compounds. The recovery of phenols was variable in function of the type of phenol and ranged from 68.6% for procyanidin B_1_ to 13% for gallic acid. Most phenols showed a recovery of more than 50%, while the recovery was slightly less than 50% in the case of esters of hydroxycinnamic acids.

The total phenol content (966.81 mg/kg) as estimated by LC-HRMS was about 5% of total phenol content measured by Folin method, indicating that the most of phenolic fraction within UG extract was of a polymeric nature [43].

### 3.3. Consumers’ Knowledge and Acceptance of Functional Foods

Results of the questionnaire about the knowledge and acceptance of functional foods are reported in Table 3. The large majority of consumers stated that they know that food plays a key role in their personal health. However, not all of them had a clear idea about what the terms “enriched” and “functional” mean; consequently, they reported neutral response (46%) to the possible positive impact of consuming functional foods on their health. Accordingly, only 21.8% of the subjects declared that functional foods are a convenient way of meeting recommended daily intakes that are difficult to reach with a conventional diet. A total 57.4% of subjects also reported a neutral response to the statement “According to my personal opinion, functional foods are too expensive given their claimed health benefit”; however, they did not seem to be ready to compromise tastiness for healthiness (only 8% agreed to compromise). Finally, only 16.8% of the subjects did not believe that fortified and/or enriched foods are healthier than traditional ones.

### 3.4. Liking Assessment under Blind, Expected, and Real Conditions

ANOVA results revealed a significant Sample effect (F_(3,1100)_ = 3.11; *p* < 0.05) on liking scores. Indeed, independently from the condition, liking decreased with increasing concentration of phenol extract and, generally, the scores fell within the labels “neither liked nor disliked” and “slightly liked” on the LAM scale. The Condition effect was also significant (F_(2,1100)_ = 16.93; *p* < 0.001), with liking scores provided in the Expected condition significantly higher (M = 56.8 ± 1.0) compared with both Blind (M = 50.9 ± 1.0) and Real conditions (M = 52.2 ± 1.1), which were comparable to each other. The Sample × Condition interaction was not significant (F_(6,1100)_ = 0.55; *p* = 0.77). Two-way ANOVA models were also computed to explore the effect of information on liking scores independently for each sample. A significant effect of the condition (Blind, Expected, and Real) on liking scores was found for samples B_0_, B_2_, and B_3_ (Table 4); Blind liking scores were significantly lower than expected, thus indicating a negative disconfirmation. A confirmation of expectations was found for sample B_1_ (B = E).

**Figure 3:**
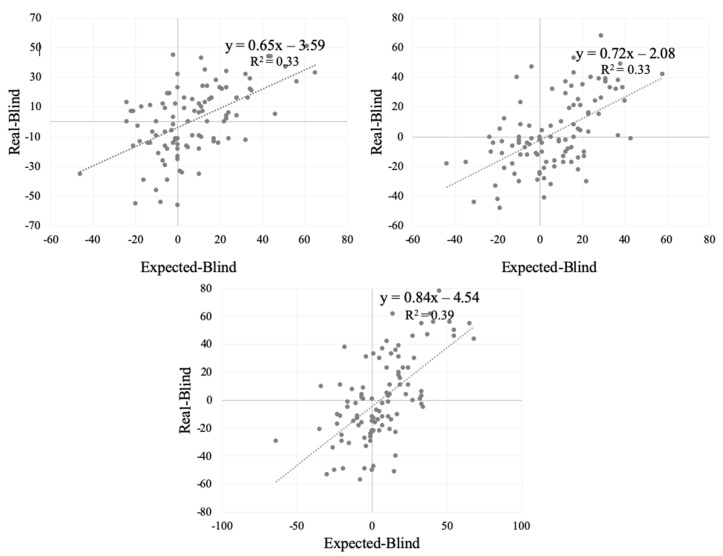
Effect of expectancy disconfirmation for B_0_, B_2_, and B_3_ samples. Linear regression plot of E–B vs. R–B scores.

Linear regressions between individual hedonic disconfirmation (E–B) and the R–B difference are reported in Figure 3. All slopes of the regression equations were positive, revealing an assimilation effect. Slope values largely differed among samples. The slope tended to be around 0.5 for product B_0_ (0.65 ± 0.1), suggesting that subjects did not assimilate the expectations because of the negative sensory properties of the product [32]. In other words, the mismatch between the Expected and Blind liking did not lead to in a positive effect of the information on the sample evaluated in the Real condition. The slope values for samples B_2_ and B_3_ were, respectively, 0.72 ± 0.1 and 0.84 ± 0.1. In these cases, the expectations generated by the information about the amount of phenols added to the beetroot samples were completely met in only some of the subjects.

### 3.5. Health and Taste Attitude Scale (HTAS)

Internal reliability as assessed by Cronbach’s alpha as well as the average of each item by gender are reported in Table 5. Cronbach’s alpha values highlighted that the internal reliability was satisfactory for each subscale [34]. Looking to the gender effect, significantly higher values were obtained by women compared with men for both “Taste” subscales. No difference by gender was found in the “Health” subscales.

A significant positive correlation (r = 0.46, *p* < 0.001) was found between “General health interest” and “Natural product interest”, and a significant negative correlation (r = −0.22, *p* < 0.05) was highlighted between General health interest and “Using food as reward”. Moreover, a significant positive correlation (r = 0.48, *p* < 0.001) was found between “Craving for sweet food” and “Using food as a reward”. 

### 3.6. Influence of Consumers’ Health and Taste Attitudes on Hedonic Responses

**Figure 4:**
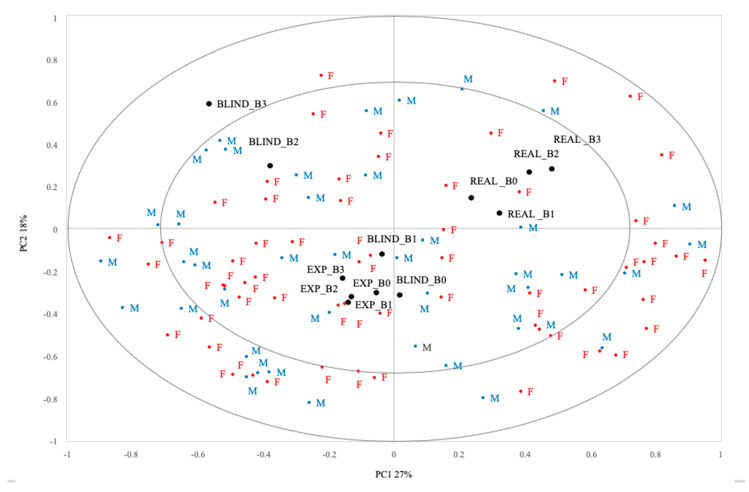
Correlation loadings with consumers and the four samples evaluated in the three conditions.

To explore health and taste attitudes influences at the individual level on the effect of the information about the phenol content of the beetroot samples on hedonic responses, a map was obtained by means of a principal-component analysis. Results are summarized in the biplot of Figure 4. The plot is an internal preference map.

Individual respondents are represented on the map by points, which can be considered end-points of vectors from the origin. The direction of the vector represents the direction of increasing personal preference for a consumer [44]. Looking at Figure 4, it is possible to observe no gender-related differences in hedonics scores, since there are no areas of the map in which participants from a specific gender are concentrated. The first component (from the left to the right of the plot) split the samples evaluated in the Real condition from the rest. Along the second dimension (from the bottom to the top), the opposition of Expected (all samples) and Blind conditions of samples B_0_ and B_1_ versus Blind condition of samples B_2_ and B_3_ is represented. 

Subjects with a negative coordinate on the second component tended to express higher expectations for the samples than subjects with a positive coordinate. Moreover, relatively lower liking expressed in the Real condition indicated a lower assimilation than others (see in the map the opposition between the samples in the Expected and Real conditions). For these subjects, the distance between liking expected and expressed in the Real condition was lower considering samples B_0_ and B_1_ than samples B_2_ and B_3_. This highlights that for these subjects, the assimilation towards expectations decreased when the phenol content in the beetroot samples increased (see the relative position on the map of the samples B_2_ and B_3_ compared to B_0_ and B_1_ in the Real condition). Subjects in the upper side of the map showed lower hedonic expectations towards beetroot puree enriched with phenols from winemaking byproducts, but a higher assimilation. 

ANOVA results revealed that these two groups of subjects (negative vs. positive values on the second component) tended to differ for the HTAS domains Natural product interest (*p* = 0.08) and Using food as a reward (*p* = 0.07), revealing that these attitudes explained the hedonic expectations for the functionalized beetroot purees. In particular, subjects on the bottom side of the map showed lower Natural product interest and higher Using food as a reward scores compared with those on the upper side. Focusing more on the group of subjects on the bottom left side of the maps and comparing their health and taste attitudes with all the other subjects, the differences previously highlighted became stronger. Indeed, subjects characterized by higher expectations presented a significantly lower Natural product interest (F_(1;99)_ = 5.58, *p* < 0.05) and significantly higher scores for Using food as a reward (F_(1;99)_ = 3.96, *p* < 0.05) compared with the other subjects.

## 4. Discussion

In the present study, a healthy enriched model food was developed by adding a phenol-rich extract obtained from unripe grapes. Thus, a winemaking byproduct was used as a value-added ingredient, with a view to moving from a linear to a circular economy. The phenol content as well as the antioxidant activity of the developed samples were investigated. Subsequently, the influence of information about the positive effects on both environment and health of the use of these ingredients in new formulations on consumers’ food acceptance and expectations were studied in relation to consumers’ health and taste attitudes.

### 4.1. Unripe Grape Extract Characterization

The TP (20.4 mg/g) content of the UG extract was similar to that observed by Kuck and Noreña [45] for extracts obtained from grape skin, cv. Bardo (*Vitis lambrusca*), microencapsulated with 10% gum arabic. These authors observed a total phenol content of 22.61 mg/g and 25.06 mg/g in the grape-skin extracts obtained by freeze-drying and spray-drying, respectively. The antioxidant activity (approximately 60 µmol/g) observed by Kuck and Noreña [45] for the grape-skin extracts was higher than that observed in the UG extracts (33.8 µmol/g). The antioxidant activity of the *Vitis lambrusca* extracts could be due to their phenol composition, which is different with respect to that of *Vitis vinifera* [46]. Moreover, the antioxidant activity of grape extracts is affected by several factors, such as variety, seasonal variation, and the maturity of the grapes [47], as well as by the extraction and drying technique [45,48]. The UG extract was stable during storage in terms of both total phenolic content and antioxidant activity. After nine months, data collected indicated that the chosen storage conditions were suitable to protect the UG phenols from degradation reactions. 

### 4.2. Total Phenol and Antioxidant Activity of Food Samples

The characteristics of a food (e.g., composition, ionic strength, pH, and physical state) can influence the antioxidant activity of natural plant phenols used to enrich or protect food from oxidation [7,49]. The percentage of UG phenols recovered (59.6%) and the significant increase of the antioxidant activity detected in the beetroot puree after the enrichment indicated that the beetroot puree contributed to both the extractability and potential biological activity retention of the UG phenols. In a similar way, an increase of both total phenol and antioxidant activity of yoghurts enriched with grape-seed extracts was obtained by Chouchouli et al. [50]. Moreover, Bekhit et al. [51] incorporated different amount of red-grape-skin extracts into a tea infusion and observed an increase of the phenolic content and antioxidant activity of the enriched infusions.

### 4.3. Consumer Responses to the New Developed Food Samples

The hedonic evaluation highlighted that all the functionalized samples were acceptable for the consumers, since liking scores always were stabilized in the middle of the LAM scale. The addition of increasing concentrations of phenol extract, independently from the tasting conditions, negatively affected hedonic responses, although with a contained effect, even at highest concentrations. Beetroot purees with higher added concentrations were less accepted compared with the control sample without the extract and the B_1_ sample. For this latter sample with 0.44 g/kg of phenol extract, consumers’ expectations were confirmed during the Real tasting condition. Therefore, phenol addition at this concentration is promising. The general decrease in hedonic responses at higher concentrations could be due to several factors. First of all, beetroot is characterized by an earthy flavour which is not always appreciated by consumers. Secondly, the lower liking scores in the B_2_ and B_3_ samples could likely be due to the specific sensory attributes that characterized the phenol extract from unripe grapes. Indeed, it has been reported that phenolic compounds can influence perceptions of bitterness and astringency when added to food and beverages [7,11]. In this context, it was recently demonstrated that the addition of the phenol extract from unripe grapes in vegetable food models (i.e., potato, beetroot and pea purees) systematically increased perceptions sourness, bitterness, and astringency [8]. These sensory attributes, which are recognized to be associated with “warning sensations”, can result in lower liking scores for phenol-rich vegetable foods [52,53]. Accordingly, it was reported that adding grape-skin powders to soft cow milk cheeses resulted in a decrease in liking scores, especially with increasing powder amounts [13]. Similarly, Lavelli et al. [14] reported that the addition of red grape skins to apple puree as a new beverage with antiglycation properties yielded a decrease in hedonic evaluations. Indeed, it was highlighted that all the sensory characteristics evaluated, especially texture, were negatively affected by the use of winemaking-derived ingredients. A negative influence on hedonic responses due to the use of waste and byproducts of wine-production chain was also shown in cereal-based products (e.g., cereal bars, noodles, and pancakes) that were enriched with grape-seed flour obtained from the pomace waste produced during winemaking [54]. 

Focusing more on the liking results obtained during the three different tasting conditions, higher liking responses were generally provided during the Expected condition, with the information delivered but without tasting the beetroot puree samples, compared with the other two conditions (Blind and Real). These results are consistent with previous research highlighting that health and nutrition information [32,55], as well as information about food sustainability [17,56] provided to consumers can positively affect expectations increasing hedonic responses. However, the health information associated with the antioxidant content did not always yield higher expected liking scores. In this context, Ares et al. [57] found that expected liking decreased when information about the antioxidants was provided to the participants evaluating functional milk desserts. Our results showed that for samples B_0_, B_2_, and B_3_, a negative disconfirmation took place, since worse ratings were reported during the Blind condition compared with ratings of the same products when information was provided. Under the Real condition, namely when the consumers received the information about nutritional and sustainability aspects as well as the samples, hedonic ratings were comparable to those from the Blind condition. Hence, this information was not enough to lead consumers to increase their hedonic scores. In order to explain the effect of the discrepancy between expectations and actual product performance on acceptance [33,58], different models (i.e., assimilation and contrast) have been proposed. Looking in more detail at the effect that the information had on hedonic scores for each sample, regression models for E–B and R–B showed all positive slopes, depicting an assimilation effect. For B_2_ and B_3_ samples in particular, the assimilation tended to be complete, but only for a proportion of subjects. This result suggested that for this segment of consumers, information about sustainability and health benefits have a positive impact on liking.

The evaluation of the impact of the taste and health attitudes on hedonic scores highlighted a group of consumers who showed higher expectations and a lower assimilation effect, compared with the other subjects. These subjects were less interested in natural products and used food as a reward more compared to the other subjects. Lower scores for Natural product interest were related to a low interest in eating food that does not contain additives or is unprocessed. Thus, we may hypothesize that for these subjects, a product modified with the addition of exogenous/external ingredients, even if natural, was more acceptable than for others. However, these subjects also gave higher scores for Using food as a reward, meaning that they could be more disposed to search for immediate rewards by consuming something really tasty. These subjects with higher expectations and low assimilation were those for whom the sensory perception had a greater weight than the information given. It cannot be excluded that under repeated tasting, sensory disconfirmations, which do not assimilate totally, can lead to a loss of product reputation through lack of reliability [59,60]. Similarly to the present finding, Ares et al. [61] reported that consumer segments with different expectations and motivations toward chocolate milk desserts enriched with different polyphenolic concentrations were found. The identified groups of consumers provided also different overall liking scores after tasting the desserts. The present findings support the assumption that consumers differently react to health and sustainability information based on their attitudes, expectations, and motivations [57]. The health and sustainability benefits may provide added value to consumers; however, these positive consequences did not compensate for consumers’ perceptions of sensory properties, which is the main driver of food choices [2,15,18]. This assumption was also corroborated from the results obtained from the questionnaire about the knowledge and acceptance of functional foods, which showed that more than half of the consumers involved in the present experiment claimed to disagree with the statement “functional foods are acceptable for me, even if they taste worse than their conventional alternative foods”. 

Contrary to the present findings, previous research highlighted an increase in hedonic responses in real tasting conditions compared with those in blind conditions. It was reported that information about the positive health effects of antioxidant components increased liking for pomegranate and green tea juices [19], unfamiliar fruit juice made with açaí [16], and juice made with different types of exotic tropical fruits (camu-camu, açaí, cajà, and umbo) [18]. The effect of information about winemaking byproduct ingredients (grape skin) on consumers’ liking was also found to have a positive effect when used for tea infusion [62]. However, literature data about information on food sustainability and organic production highlighted mixed results [17,63]. The discrepancy found in the literature might be ascribed to the different natures of the food products tested, and also to the way in which the information about sustainability and health consequences was provided. Moreover, the present findings also highlighted an information effect for the sample B_0_, which did not contain the phenol extract, but this could be associated with the fact that consumers might have perceived the beetroot puree as a healthy product in itself, besides the enrichment. 

## 5. Conclusions

In conclusion, the development of new phenol-enriched food using unripe grapes discarded during thinning operations is challenging due to the sensory specific attributes of this ingredient. However, it is also promising, since all the samples were generally accepted by the consumers and they presented a phenol amount that remained stable over time and that could have positive health effect following consumption. It can be also hypothesized that the model food evaluated herein could be added to other ingredients in a final gastronomic preparation, which could significantly improve its hedonic performance. Finally, the influence of health properties and sustainability-related production information seems to be effective depending on consumer attitudes towards health- and taste-related issues.

## Figures and Tables

**Table 1:** Total phenol (TP) content and antioxidant activity (AA) of the unripe grape (UG) extract during storage. The data are the mean of three replicates ± standard deviation. The superscript letter indicates that no significant differences were observed (*p* > 0.05).

Months	TP (mg/g)	AA (µmol TEAC/g)
0	20.40 ± 0.9 ^a^	33.80 ± 1.8 ^a^
3	20.35 ± 0.8 ^a^	33.95 ± 1.9 ^a^
6	20.42 ± 0.9 ^a^	33.78 ± 1.6 ^a^
9	20.38 ± 1.0 ^a^	33.91 ± 1.5 ^a^

**Table 2:** Phenol composition of the UG extract stock solution (6.81 g/L of phenols), UG extract (powder), beetroot puree, and beetroot puree functionalized with 1.93 g/kg of phenols from the UG extract.

Compound	UG Extract Stock Solution (mg/mL)	UG Extract (mg/kg)	UG Phenol Added to Beetroot Puree (mg/kg)	Beetroot Puree (mg/kg)	Functionalized Beetroot Puree (mg/kg)	Recovery (%)
Caffeic acid	0.46 ± 0.01	12.11	1.63	nd	1.08 ± 0.00	66.3
Coumaric acid	0.79 ± 0.02	20.86	2.81	0.08 ± 0.00	1.79 ± 0.01	61.9
Ferulic acid	0.19 ± 0.02	4.93	0.66	2.37 ± 0.05	1.73 ± 0.16	57
Caftaric acid	27.6 ± 1.24	730	98.2	0.05 ± 0.00	47.9 ± 0.18	48.7
Coutaric acid	1.33 ± 0.04	35.13	4.73	nd	2.25 ± 0.02	47.6
Fertaric acid	2.00 ± 0.08	52.82	7.11	0.38 ± 0.07	3.37 ± 0.01	45.1
Gallic acid	0.07 ± 0.00	1.92	0.26	nd	0.03 ± 0.00	13
Isorhamnetin	0.05 ± 0.00	1.36	0.18	nd	0.05 ± 0.00	26.8
Kaempferol	0.03 ± 0.00	0.78	0.1	nd	0.06 ± 0.00	57.5
Kaempferol-3 *O*-glucoside	0.02 ± 0.00	0.48	0.07	nd	nd	-
Myricetin	0.13 ± 0.00	3.56	0.48	0.10 ± 0.00	0.39 ± 0.00	66.6
Myricetin-*O*-hexoside	nd	nd	-	0.43 ± 0.00	0.43 ± 0.00	100
Quercetin	0.54 ± 0.01	14.33	1.93	nd	1.31 ± 0.01	67.9
Quercetin-3-*O*-hexoside	0.05 ± 0.00	1.27	0.17	nd	nd	-
(+)-Catechin	0.54 ± 0.03	14.16	1.91	nd	1.25 ± 0.01	65.5
(−)-Epicatechin	0.35 ± 0.01	9.23	1.24	nd	0.71 ± 0.00	57.4
Procyanidin B_1_	0.18 ± 0.00	4.68	0.63	nd	0.43 ± 0.00	68.6
Procyanidin B_2_	0.36 ± 0.01	9.48	1.28	nd	0.76 ± 0.07	59.2
Trans-resveratrol	1.18 ± 0.06	31.12	4.19	nd	2.18 ± 0.01	52.1
2-*S*-Glutathionyl caftaric acid	0.71 ± 0.02	18.63	2.51	nd	0.68 ± 0.13	27.2
Sum	36.58	966.81	130.13	3.41	66.63	51.2

Data are expressed as mean ± standard deviation (*n* = 3); nd: not detected.

**Table 3:** Knowledge and acceptance of functional foods—frequency distributions of Likert-scaled variables (%, *n* = 101).

Item Pool	Strongly Disagree	Disagree	Neutral	Agree	Strongly Agree
1. Food plays an important role for my personal health	-	-	-	14.9	85.1
2. I know enriched and functional foods	6.9	9.9	40.6	23.8	19.8
3. Functional foods are likely to have a beneficial impact on my personal health	3	4	46.5	26.7	19.8
4. Functional foods are a convenient way of meeting recommended daily intakes which I would never meet with my conventional diet	22.8	15.8	39.6	15.9	5.9
5. According to my personal opinion, functional foods are too expensive given their claimed health benefit	8.9	21.8	57.4	11.9	-
6. Functional foods are acceptable for me, even if they taste worse than their conventional alternative foods	39.6	22.8	29.7	8	-
7. I do not believe that fortified and or enriched foods are considered healthier than traditional ones	25.7	22.8	34.7	16.8	-

**Table 4:** Mean liking scores provided for each beetroot sample in the three different conditions. Different superscript letters in a row indicate significant differences according to post hoc test.

Samples	Condition			
	Blind (B)	Expected (E)	Real (R)	F
B_0_	51.2 ^a^ ± 1.5	58.8 ^b^ ± 1.6	52.7 ^a^ ± 1.5	6.95 **
B_1_	53.6 ^a^ ± 1.4	56.6 ^a^ ± 1.5	53.9 ^a^ ± 1.5	1.45 n.s.
B_2_	49.7 ^a^ ± 1.6	56.2 ^b^ ± 1.6	52.3 ^a^ ± 1.6	4.76 *
B_3_	48.9 ^a^ ± 1.9	55.4 ^b^ ± 1.8	49.9 ^a^ ± 1.8	3.81 *

* *p* < 0.05; **p < 0.01.

**Table 5:** HTSA subscales means by gender and Cronbach’s alpha values.

HTSA	Cronbach’s Alpha	Mean ± SEM		
		women	men	
*Health subscale*				
General health interest	0.80	4.7 ± 0.1	4.9 ± 0.1	n.s.
Light products interest	0.79	3.9 ± 0.1	3.9 ± 0.1	n.s.
Natural product interest	0.82	3.5 ± 0.2	3.9 ± 0.2	n.s.
*Taste subscale*				
Craving for sweet foods	0.88	4.7 ± 0.2	5.4 ± 0.2	**
Using food as a reward	0.81	4.0 ± 0.2	4.8 ± 0.2	**

** *p* < 0.01.
